# Principles underlying the design of "The Number Race", an adaptive computer game for remediation of dyscalculia

**DOI:** 10.1186/1744-9081-2-19

**Published:** 2006-05-30

**Authors:** Anna J Wilson, Stanislas Dehaene, Philippe Pinel, Susannah K Revkin, Laurent Cohen, David Cohen

**Affiliations:** 1 INSERM-CEA Unit 562 « Cognitive Neuroimaging », Service Hospitalier Frédéric Joliot, CEA-DRM-DSV, 91401 Orsay, France; 2 Collège de France, 11 place Marcelin Berthelot, 75231 Paris Cedex 05, France; 3 Service de Neurologie, Hôpital de la Pitié-Salpêtrière, AP-HP, 47 bd de l'Hôpital, 75013, Paris, France; 4 Department of Child and Adolescent Psychiatry, Université Pierre et Marie Curie, Laboratoire CNRS "Du comportement et de la cognition", Hôpital Pitié-Salpêtrière, AP-HP, 47 bd de l'Hôpital, 75013, Paris, France

## Abstract

**Background:**

Adaptive game software has been successful in remediation of dyslexia. Here we describe the cognitive and algorithmic principles underlying the development of similar software for dyscalculia. Our software is based on current understanding of the cerebral representation of number and the hypotheses that dyscalculia is due to a "core deficit" in number sense or in the link between number sense and symbolic number representations.

**Methods:**

"The Number Race" software trains children on an entertaining numerical comparison task, by presenting problems adapted to the performance level of the individual child. We report full mathematical specifications of the algorithm used, which relies on an internal model of the child's knowledge in a multidimensional "learning space" consisting of three difficulty dimensions: numerical distance, response deadline, and conceptual complexity (from non-symbolic numerosity processing to increasingly complex symbolic operations).

**Results:**

The performance of the software was evaluated both by mathematical simulations and by five weeks of use by nine children with mathematical learning difficulties. The results indicate that the software adapts well to varying levels of initial knowledge and learning speeds. Feedback from children, parents and teachers was positive. A companion article [1] describes the evolution of number sense and arithmetic scores before and after training.

**Conclusion:**

The software, open-source and freely available online, is designed for learning disabled children aged 5–8, and may also be useful for general instruction of normal preschool children. The learning algorithm reported is highly general, and may be applied in other domains.

## Background

In the last few decades, cognitive neuroscience research has revealed that developing cognitive brain systems appear to show considerable plasticity in response to early brain lesions or neuronal disorganization. Although many of the constraints and critical periods for plasticity remain to be defined, this finding has already started to be applied to education, particularly to the field of learning disabilities. To provide the intensive training necessary to induce long-lasting brain changes, researchers have designed "adaptive games", i.e. computer programs which use algorithms to adapt to an individual child's ability and provide intensive training in an entertaining context. This approach has now been successfully used to improve language and reading performance in children with specific language impairment (SLI) and dyslexia [2-4]. Functional magnetic resonance imaging (fMRI) has shown noticeable changes in brain activity associated with adaptive game remediation, including a partial restoration of normal activation in reading-related areas [4].

Adaptive game software has the potential to maintain the difficulty of an educational task within the "zone of proximal development" [5], minimizing failure whilst maintaining adequate difficulty, thus providing a presumably ideal level of cognitive stimulation needed for progress. The use of computer aided instruction also allows us to capitalize on the fascination that children have for computer games, which makes it easier to provide intensive training on exercises which might otherwise become boring for them. In modern societies, computers have become so ubiquitous that computer-aided instruction is now low-cost, and can be used in either the home or the school environment.

In this article we report the first development of an adaptive game for remediation of dyscalculia, inspired by cognitive neuroscience research. Dyscalculia, or mathematical learning disability, is thought to have a prevalence of 3–6% [6-9], similar to that of dyslexia, but is extremely under-researched in comparison, despite the grave professional and societal consequences which it entails [10]. It is unknown whether dyscalculia can be successfully remediated, or whether in general the brain circuits involved in numerical cognition show developmental plasticity [11]. However previous success has been shown in school-based intervention with children at-risk for low mathematical achievement [12,13], as well as in neuropsychological training in adults with acquired dyscalculia [14]. Inspired by this work, and that in the dyslexia field, our aim was to develop a fully automatized computerized adaptive game that would be entertaining, and yet would inconspicuously provide intensive training in number sense.

The article is structured as follows. We first outline the key instructional principles used in designing the software. We then discuss the software design and development in detail. Finally, we report on the performance of the software both in mathematical simulations and in five weeks of use by nine children with mathematics learning difficulties. In a companion article [1], we present a first open-trial study in full detail, including results of number sense and arithmetic tests before and after training. This article also discusses dyscalculia in more depth.

## Instructional principles

The design of the software was based on several instructional principles relevant to the remediation of developmental dyscalculia, although these principles may be equally pertinent to the instruction of mathematics for younger non-dyscalculic children. We mention possible symptoms and causes of dyscalculia here only briefly and where relevant to particular design features.

### 1. Enhancing number sense

Our most important design principle was that of enhancing quantity representation, or number sense. We now know that one of the most fundamental aspects of numerical cognition is the ability to represent and manipulate approximate numerical quantities in a non-verbal format [15-18]. This ability remains at the core of many numerical tasks, even once symbolic representations such as Arabic digits have been learned. We and others have suggested that dyscalculia may involve an impairment in quantity representation or its access via symbolic representations (the "core deficit" theory) [19-24].

In order to enhance number sense, we firstly selected number comparison as the primary task of the software. Number comparison is a simple task which draws heavily on quantity representation [15,25-27], and which produces activity in the area of the brain thought to underlie a neuronal code for numerical quantity, the horizontal intra-parietal sulcus (HIPS) [28,29]. The difficulty of the task and degree of associated brain activity is modulated by numerical distance in adults and children [30,31]. Dyscalculic children and children who are at risk for mathematical under-achievement perform slowly or inaccurately in numerical comparison [13,22,32-34]. Our comparison task included varying levels of numerical distance, thus allowing the software to adapt to the current level of precision of the child's quantity representation. We also included an adaptable response deadline to encourage faster, increasingly automatic access to quantity representation.

The software was also designed to emphasize the association between representations of number and space, which are known to be closely linked [15,27,35]. One previous highly successful number sense intervention achieved this by capitalizing on the key features of board games, in which the number/space link is concretized as playing pieces are moved along the board; the distance of their moves being enumerated or estimated numerically by children [12,13].

### 2. Cementing the links between representations of number

As mentioned above, a core deficit in number sense could be caused by a deficit in number sense itself, or in the links between this representation and learned symbolic representations of number. Our second instructional principle was therefore to cement links between non-verbal quantity representation and other developing symbolic representations of numbers, such Arabic numerals or number words. This was achieved by the following two methods: a) a scaffolding procedure which required children to rely increasingly on symbolic representations in order to perform the numerical comparison task, and b) a "repeated association" feature which concurrently presented all three number formats after the child had made their response to this task.

### 3. Conceptualizing and automatizing arithmetic

A third instructional principle was to increase understanding of and fluency of access to very basic addition and subtraction facts. Dyscalculic children tend to show a developmental delay in procedures used to calculate simple addition and subtraction sums, as well as fact retrieval deficits. In particular, they tend to use laborious finger counting procedures when other children have already switching to verbal counting, decomposition and, later, memory retrieval procedures. [36-38]. This delay seems to be extremely persistent, with a series of longitudinal studies by Ostad [39,40] showing that it lasts at least up until 5th grade for addition and 7th grade for subtraction. It could be that dyscalculic children are slow to develop these more advanced procedures and to memorize facts because of a difficulty understanding the meaning of numbers and the operations concerned. At higher levels of difficulty of the software we therefore included small addition and subtraction facts which children had to solve prior to making a numerical comparison. These operations were reinforced with concrete representations of sets of objects undergoing the corresponding transformations. The aforementioned adaptable response deadline added speed pressure to these arithmetic tasks, thus progressively encouraging the use of more advanced procedures such as fact retrieval or decomposition.

### 4. Maximizing motivation

Finally, a fourth important instructional principle common to all adaptive games was to maintain attention and motivation by providing sufficient positive reinforcement. This was achieved by the adaptive algorithm itself, which was programmed to continuously adapt task difficulty in order to maintain performance at 75% correct. In addition to maximizing motivation, this "rewarding" environment may help with other problems which can be associated with dyscalculia. For instance ADHD (attention deficit and hyperactivity disorder) appears to be highly associated with dyscalculia [7], and children with this disorder may benefit from a high-reinforcement environment [41]. It addition, a high reward rate may help reduce the considerable anxiety that many dyscalculic children experience when exposed to mathematics [42], by associating mathematical activity with a positive emotional state.

### Potential for general instructional use

As mentioned earlier, although the initial conception and testing of the remediation was for and with dyscalculic children, it is likely that these same principles would aid in the development of number sense for non-dyscalculic children, at an earlier age. Thus there is potential for this software to be used with children who are at risk for under-achievement due to socioeconomic status, or with children who are just learning the meaning of number symbols. In the next year, in association with collaborators, we will be testing the software in normal kindergarten children to see if it can accelerate their learning.

## Methods

"The Number Race" software was programmed by Anna Wilson, over the period of one year. It is written in Java and is thus multi-platform. The current version is available in French and German; however English, Swedish, and Finnish translations are planned. The software is under a GNU license, and is thus open source and freely available. It can be downloaded from our lab website [43].

### Overall game design

**Figure 1:**
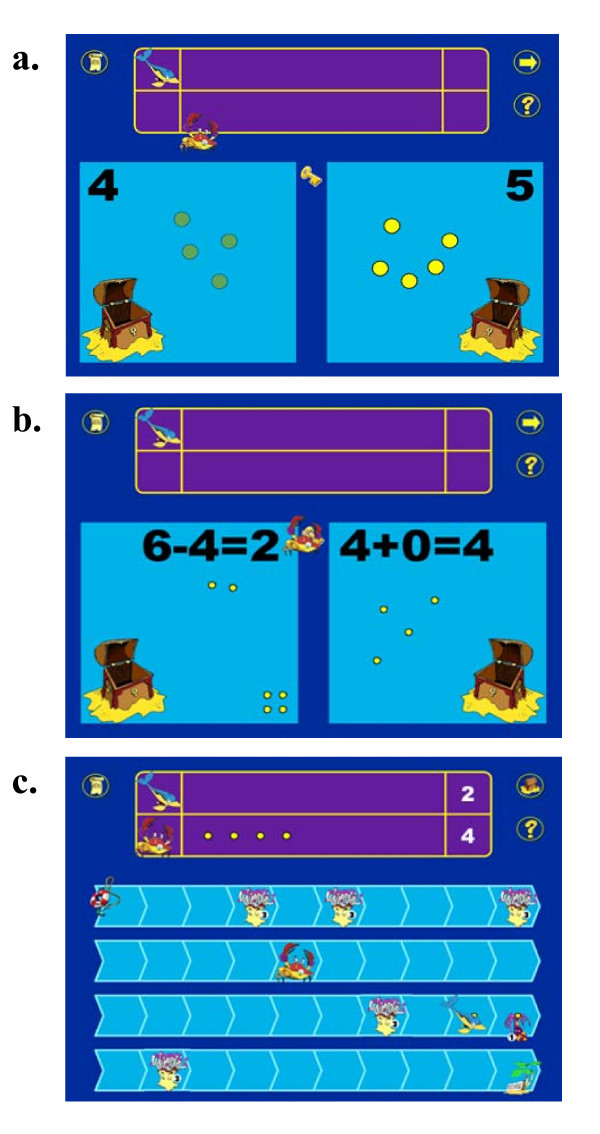
**Screen shots from the "The Number Race" rehabilitation software**. a. Sample comparison screen. The child plays the character of the dolphin, and has to choose the larger of two numerosities, before her competitor (the crab) arrives at the key and steals the larger quantity. b. Another sample comparison screen, taken at a higher difficulty level on the "complexity" dimension where addition and subtraction are required to make a correct comparison. The screen shows how operations are concretized by corresponding operations on sets of objects, after one of the characters wins (in this case the competitor). c. Sample board screen. After each comparison, the child uses tokens won to move a corresponding number of squares on the game board, where she must avoid landing on hazards (here depicted by anemones). Once she arrives at the end of the board, she wins a "reward" fish to add to her collection. Winning enough of these rewards unlocks access to the next character.

There are two main screens in the game. On the "comparison screen" (Figure 1a), children carry out a numerical comparison task, choosing the larger of two quantities of treasure (ranging from 1 to 9). This is the primary task whose difficulty is manipulated by the adaptive algorithm (see following section for full explanation). On some trials the task has a response deadline; the competitor character which children play against (controlled by the computer) moves to take the largest quantity if the child does not respond in time. In higher levels, the child may have to add or subtract in order to make the comparison (Figure 1b). Prior to the child's choice, the quantities can be presented in non-symbolic format (sets of "gold pieces", which are controlled for density or luminance), in symbolic Arabic format (digits), in symbolic verbal format (spoken number words), or in a combination of these formats. After they make a choice, they are presented with all three of these formats (e.g. they see five coins, the digit 5, and hear "you chose five"), and they also receive feedback on who has "won" this turn (e.g. "You've got the most!").

After choosing on the comparison screen, children move to the "board screen" (Figure 1c) where they use their set of gold pieces to advance in a race against the competitor character. They have to move their character (and then their competitor) by the same number of squares as each has gold pieces. They do this by clicking the gold pieces onto the board in one-to-one correspondence. This can be done one at a time, or by counting and clicking only on the correct end square, in which case the pieces move all at once. The number of squares is counted out aloud by the computer as the child clicks the gold pieces in place, and again as their character moves. Children receive feedback on the relative position of the two characters (e.g. their competitor says "I'm way behind!", or "Oh no! You've overtaken me!"). At higher levels, they have to try and avoid hazards, which appear at random on some board squares and cause characters to go backwards when landed on.

When children reach the end of the board before their computer-driven competitor does, they collect a reward. After enough rewards are collected, they can unlock a new character to play with. The new character has new animations, but the core game tasks remain exactly the same.

### Adaptive dimensions

We used a multidimensional learning algorithm to constantly adapt the difficulty of the program to the child's performance level. Adaptation was implemented using three dimensions of difficulty, which were based on our instructional principles and our knowledge of the key determinants of performance in basic numerical cognition in adults and children. Below we describe these dimensions conceptually, and in the following section, mathematically.

1. The first dimension, "distance", increases difficulty of the numerical comparison by decreasing the numerical distance (as measured by the Weber ratio) between the two compared quantities. This dimension is designed to adapt to the precision of the children's quantity representation, and to push children to progressively increase this precision.

2. The second dimension, "speed", implements an increasingly short deadline by which the child must respond. This is designed to increase speed and automaticity of to quantity representations, and to encourage more efficient calculation and eventually memory recall of simple number facts. At the lower end of this dimension, there is no deadline, so that if children are particularly slow at a task, they will still be able to succeed.

3. The third dimension, "conceptual complexity", is a composite dimension which is designed to move children along a pedagogical progression which teaches them about number symbols and elementary arithmetic. Difficulty is increased in two ways: 1) by decreasing the ratio of non-symbolic to symbolic information available to make a choice between the two quantities on the "choice screen", and 2) by introducing addition and subtraction at higher levels. These steps were designed to cement links between symbolic and non-symbolic representations of number, and to increase understanding and of and fluency of access to simple arithmetical facts. However the dimension includes some other aspects, such as restricting magnitude range at times, and adding hazards to the board (see Table 1 for full details).

**Table 1:** Conceptual complexity dimension levels

Level	Format given before choice:			Range restriction? (numbers 1–5 only)	Dot fading present? (duration)	Hazards present?	Addition required?	Subtraction required?	Instructional goal
							
	Non-symbolic (dot clouds)	Symbolic: Verbal (spoken numbers)	Symbolic: Arabic (digits)						
1	Yes	No	No	Yes	No	No	No	No	Attention to and processing of small non-symbolic quantities
2	Yes	No	No	No	No	No	No	No	Attention to and processing of large non-symbolic quantities
3	Yes	Yes	Yes	Yes	No	No	No	No	Link small non-symbolic quantities to symbolic codes
4	Yes	Yes	Yes	No	No	No	No	No	Link large non-symbolic quantities to symbolic codes
5	Yes	Yes	Yes	No	Yes (4 sec)	No	No	No	Increase reliance on symbolic codes
6	Yes	Yes	Yes	No	Yes (1 sec)	No	No	No	Further increase reliance on symbolic codes
7	No	Yes	Yes	No	No	No	No	No	Require complete reliance on symbolic codes
8	No	No	Yes	No	No	No	No	No	Require complete reliance on Arabic code
9	No	No	Yes	No	No	Yes	No	No	Attention towards exact quantity
10	No	No	Yes	Yes	No	Yes	Yes	No	Comprehension and fluency of small addition problems
11	No	No	Yes	No	No	Yes	Yes	No	Comprehension and fluency of larger addition problems
12	No	No	Yes	Yes	No	Yes	No	Yes	Comprehension and fluency of small subtraction problems
13	No	No	Yes	No	No	Yes	No	Yes	Comprehension and fluency of larger subtraction problems
14	No	No	Yes	No	No	Yes	Yes	Yes	Distinguishing between addition and subtraction

Notes.

"Range restriction" means that the range of quantities presented was from 1–5.

"Dot fading" means that the collections of gold pieces were faded from view in the space of either 1 or 4 seconds, gradually helping to introduce a reliance on symbolic codes.

"Hazards" means that there were new anemone hazards placed on the game board, which children had to try and avoid, encouraging a focus on exact quantity

In addition trials, instead of presenting a simple quantity, an addition problem with a sum of up to 9 was presented on one side of the screen.

In subtraction trials, the problems had operands of 9 or below, and thus a result of 8 or below.

### A multidimensional adaptive algorithm

The combination of the three above dimensions can be seen as constituting a "learning space". If we represent difficulty on each dimension using the interval zero to one, this learning space can be visualized as a cube. Children can be presented with a problem at any point in this space, and will have different probabilities of success for problems at different points. For instance a relatively easy problem which is at 0.1 difficulty on all three dimensions might have a high probability of success, whereas a more difficult problem located at 0.9 on all dimensions might have a low probability of success. Thus, the three-dimensional matrix giving the probability of success at each point in the learning space can be thought of as an operational definition of the current knowledge of the child. Different children can be expected to show different probability of success matrices. For instance, imagine a child who has little difficulty with numerical distance but great difficulty in responding fast, and who, whilst understanding Arabic digits, has not fully mastered addition or subtraction. We could imagine that the "knowledge area" of this child might consist of a rectangular volume of high probability of success which extends over most of the distance axis, little of the speed axis, and half of the "complexity" axis, whereas the rest of the area would be occupied by low probability of success. As the child learns, the high probability of success area ought to expand to occupy more of the total knowledge space.

How can an adaptive algorithm be written to model knowledge and learning in a multidimensional learning space such as this? The task of this algorithm is to estimate what the knowledge space looks like for each child, and to present children with problems on which the child will perform well most but not all of the time, i.e. problems in their "zone of proximal learning". The algorithm must be able to continuously update its representation of the child's knowledge space as he or she learns.

We solved this problem by using a probabilistic exploration of the knowledge space. The algorithm samples points within the space and uses the child's response to these problems to build an interpolated model of the entire knowledge space. Each turn of the game, the algorithm calculates performance over the last 20 turns, and selects a problem in the space which it estimates to be at the difficulty level required to maintain performance at 75% correct.

### Algorithm specifications

#### Goal

The purpose of the algorithm is to adapt to the performance of a learner in an n-dimensional problem space, composed of continuous or discrete dimensions, maintaining their success rate close to a pre-specified fixed level. This is achieved by estimating the learner's current knowledge in a discrete model of this problem space, and by using this representation to present the learner with problems at the level of difficulty required to maintain the desired success rate.

#### Modeling of knowledge

If *n *= the number of dimensions along which problem difficulty may vary, then we represent the estimated probability of success on all possible problem types in an *n *dimensional matrix (*K*), of size *m *on each dimension (the "knowledge matrix"), which discretizes the learning space cube of size [0,1]^n^. The value of *m *determines the "resolution" of the algorithm, and must be sufficiently large to allow adequate representation of the knowledge space, but not so large as to excessively slow computation. In our implementation, *n *= 3 and *m *= 20, hence *K *is a 20 × 20 × 20 matrix. Assume zero initial knowledge, by initializing all elements to the chance success rate (*c*).

#### Selection of problem difficulty

Let: *S *= average desired success rate; *α *= learning rate adjustment factor; and *σ *= standard deviation of scatter function. On a given trial, in order to ensure that p(correct response) ~ *S*, use the following procedure:

1. Calculate the desired success (*s*) for this trial as follows:

a. For the first five trials, *s = S*.

b. For all other trials, let *r *= the average success on the last 20 (or less) trials. *s = S - α *(*r *- *S*)

Note: low values of *α *will reduce large fluctuations in *s*, but will also result in slower adaptation to the learner's performance.

2. Identify the point (or points) in the knowledge space matrix (*K*) whose value is closest to that of *s*.

Note: an alternative method is to use a tolerance value *t *(e.g. 0.05), and identify all points whose value lies in the interval *s ± t*. If no points are found, increment *t *by an increment value *i*, (e.g. 0.01), and repeat.

3. Pick one of these matrix points at random, and convert it to a vector on the difficulty scale (i.e. in the range [0,1]^n^).

4. Sample the final problem difficulty (a vector, *d*, also in the range [0,1]^n^) from a Gaussian distribution whose mean is at the point selected in the previous step, and whose standard deviation is *σ*.

5. Present a problem at difficulty level *d *to the child, and collect the child's response (correct or incorrect)

#### Updating of the knowledge space

The observation that the learner succeeded or failed at problem *d *allows us to update the estimated knowledge matrix, *K*. Let: *ω *= adjustment rate; and *g *= generalizing distance (which is proportional to *m*). After each trial, use the final problem difficulty vector (*d*) determined in step (4) above, and the resulting Boolean success (*γ *= 1 for success, 0 for failure) to update the knowledge matrix (*K*), as follows:

1. Generalize weakly to neighboring problems:

a. Let *v *= the difference vector between *d *and each location in *K*.

b. Let the distance factor, *β*, = . (Note: |*v*|_1 _= the L1 norm of *v*, or the sum of its elements.)

c. For all elements of *K *for which |*v*|_1 _≤ *g*, adjust the estimated knowledge as follows: *k*_*current *_= (1-*ωβ*)*k*_*previous *_+ *ωβγ*

2. In case of success, generalize to all simpler problems:

a. Set *β *= 0.5

b. For all elements of *K *for which all indices are smaller than or equal to the corresponding elements of *d*, adjust the estimated knowledge as follows:

*k*_*current *_= (1 - *ωβ*)*k*_*previous *_+ *ωβγ*

3. In case of failure, generalize to all harder problems:

a. Set *β *= 0.5

b. For all elements of *K *for which all indices are larger than or equal to the corresponding elements of *d*, adjust the estimated knowledge as follows:

*k*_*current *_= (1 - *ωβ*)*k*_*previous *_+ 0.5*ωβγ*

Note: The following parameter values were used in our implementation:

*n *= 3, *m *= 20, *c *= 0.5, *S *= 0.75, *t *= 0.05, *i *= 0.02, *α *= 0.7, *σ *= 0.05, *ω *= 0.5, *g *= 4

#### Operational definition of our three adaptive dimensions

As above, let *d *be a point in knowledge space, defined by its three coordinates (*d*_*s*_*, d*_*d*_*, d*_*n*_), where *d*_*s *_= difficulty for speed dimension, *d*_*d *_= difficulty for distance dimension and *d*_*c *_= difficulty for complexity dimension. We now describe how particular values of these coordinates (the *d*_*i*_) are converted into actual problems presented to children. Note that we assume that the *d*_*i *_are real numbers in the interval [0,1]. As described in the previous section, the knowledge space is discretized in *m *steps. In that case, if the *δ*_*i *_∈ {1, *m*} are the indices of the matrix K, one can let *d*_*i *_= (*δ*_*i *_- 1)/(*m *- 1)

##### Distance dimension

The difficulty on the distance dimension *d*_*d*_, is used to select the pair of numbers *x *and *y *which are presented to the child for number comparison. We suppose that *x *is the larger number of the pair, and choose *x *such that

*x *= floor((*x*_max _- *x*_min _+1)*α *+ *x*_min_),

where *x*_max _= the largest possible value of *x *(which varies with the difficulty on the complexity dimension; see below), *x*_min _= the smallest possible value of *x *(always 2 in our implementation), and *α *= a value randomly picked from a uniform distribution in [0,1].

Then choose *y *such that

y = min(floor(),(*x *- 1))

Apart from eliminating rounding error, this equation ensures that log *y *= log *x *+ *δ*, where *δ *= (*d*_*d *_- 1).log *R*, and *R *= the maximum desired ratio of *x/y*, with the constraints that *y *≥ 1, *y *<*x*. In our implementation, we used *R *= 2.

##### Speed dimension

The deadline duration (*v*) which is used on a given trial is determined by the following functions:

if *d*_*s *_<*α*, *v *= infinity

else if *d*_*s *_≥ *α*, *v *=  + *v*_min_

where *v*_*min *_= asymptotic minimum deadline duration (in seconds)

*v*_*max *_= maximum deadline duration (in seconds)

*r *= deadline decrease rate

*α *= difficulty level at which deadline begins to be instituted

and     

We used the following parameters in our software: *v*_*min *_= 0.25, *v*_*max *_= 10, *r *= 0.001, *α *= 0.3.

##### Conceptual complexity dimension

The complexity level (*l*) which is used on a given trial is determined by the following equation, where *n *is the total number of levels:

*l *= Floor(*d*_*n*_·*n*) + 1

We used 14 complexity levels, whose detailed description is provided in Table 1.

## Results

### Validation by simulation

We tested the design of our algorithm by developing a Matlab model which simulates a child playing the game. This allowed us to run the algorithm through many tests at a high speed without being slowed by the graphical aspects of the game in Java, and to easily produce graphs of the algorithm and simulated children's performance.

There are many ways in which a child's learning could be simulated, and several were successfully tried. Here, we report solely on our simplest attempt, which modeled the child's knowledge as a rectangular volume of increasing size. Our child simulator module worked by representing the child's knowledge at a given moment by a knowledge matrix representing the success probability at each point in learning space, as in the adaptive algorithm. Probability of success at a given problem was given by a sigmoid function of the distance between the problem's location and the subject's "knowledge threshold", a set of three coordinates which specified the location in knowledge space of the corner of the subject's rectangular zone of high knowledge. This could move up from trial to trial, thus simulating learning. The speed of learning was assumed to be a function of the derivative of the sigmoid (i.e. learning occurs at the fastest rate when the child is tested on problems that are slightly harder than his/her current ability). We assumed that the rate of learning could be different in different dimensions.

#### Simulations with a fixed level of the child's knowledge

**Figure 2:**
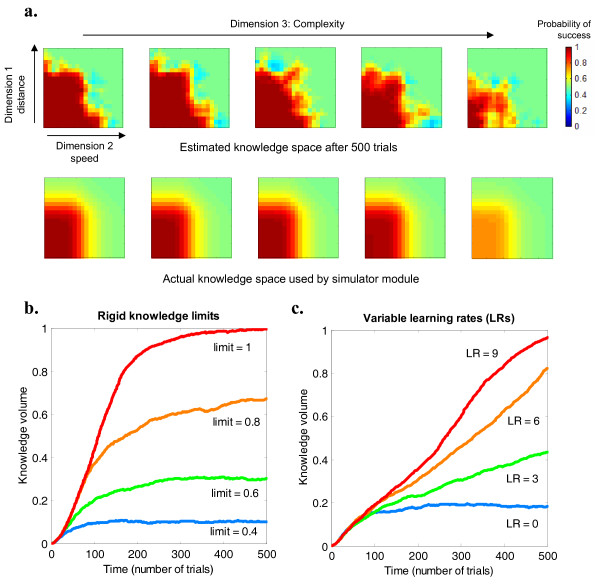
**Simulations of the adaptive algorithm and measures of learning**. a. Knowledge space estimated by the algorithm (top) after 500 simulated trials, shown as five "slices" through the three-dimensional knowledge space cube. Red represents high probability of success, blue high probability of failure, green background = chance level (50%). The estimated knowledge space resembles the actual knowledge surface (bottom) that was used by the simulator module. b. Estimated knowledge volume as a function of the number of trials. Simulated children had a knowledge limit of a rectangular cube of a particular size starting at the origin beyond which they could not progress. It can be seen that the algorithm quickly converges towards the appropriate knowledge volume (approximately the cube root of the imposed limit). c. Here, simulated children had no knowledge limit, but variable learning rates. The program tracked the progressive increase in the knowledge volume. (Knowledge started at 0.5 on each dimension).

The goal of this first simulation was to study the performance of our algorithm with a simulated child who has a fixed level of knowledge (although not the same value on all dimensions), and zero learning rate. We examined whether the algorithm was able to develop an adequately accurate model of the child's ability. Figure 2a shows the final estimated knowledge space produced by the learning algorithm (after 500 trials), which models fairly well the shape of the knowledge space defined in the simulator module. Figure 2b shows a different type of measure, the "knowledge volume" (defined as the proportion of knowledge space with an estimated probability of success greater than 75%). Here we can see that after about 100 trials the algorithm clearly distinguishes between children with different levels of fixed knowledge.

#### Simulations of the evolution of the child's knowledge

The goal of the second simulation was to investigate how well the algorithm could respond to different rates of learning. Thus the simulation was run with simulated children who started with the same level of knowledge, but had different learning rates for different dimensions. Figure 2c shows the change in knowledge volume observed. We can see that the rate of increase in knowledge volume is a function of the learning rate, especially after around 120 trials. Thus, the algorithm successfully adjusts to children's changes in learning rate. Further simulation (not shown) confirmed this capacity to track such changes even when the learning speed differed across the three different dimensions.

### Validation by testing in children

We tested the software in a 5-week open-trial study with nine children who had mathematics learning difficulties. The method and results of this study are fully described in the accompanying paper [1]; here we describe only the performance of the software itself. Children used the software for half an hour per day, four days a week, over a five week period.

#### Analyses

**Figure 3:**
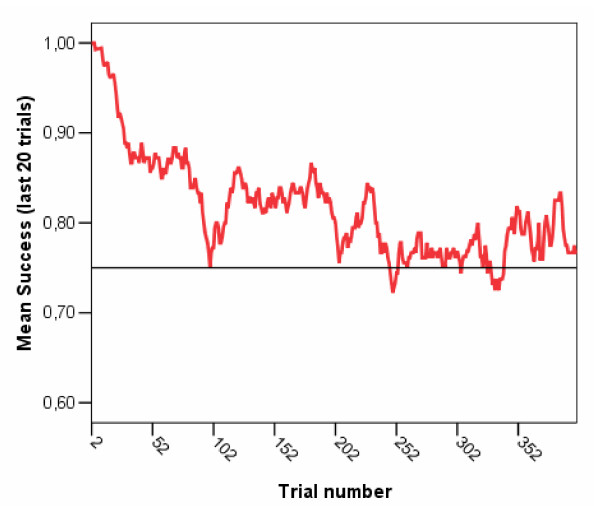
**Performance of the adaptative algorithm in ensuring a defined level of success**. Children's average mean success at each trial in the software study (measured as a running average of the last 20 trials for each child, and averaged across all nine participants). This gives an indication of how well the software adapted to children's performance, i.e. how well it stayed at the desired mean success rate of 75%. We can see that for the first half of the remediation, mean success was higher than 75%, but it eventually converged close to this value.

We analyzed children's data from the software in order to assess whether it had performed as expected. Overall, the program was fairly successful in maintaining a challenging yet rewarding level of play. Following an initial period of high success, children's performance quickly entered the 80–90% range, and eventually all children stabilized at around 75% across trials (see Figure 3). However this stabilization at the desired success rate took longer than expected, not occurring until around 250 trials. This may have been caused by fact that the algorithm assumed zero starting knowledge for each child, and then took some time to adapt up to his or her ability level. In a revised version of the software, this problem has been addressed (see below).

**Figure 4:**
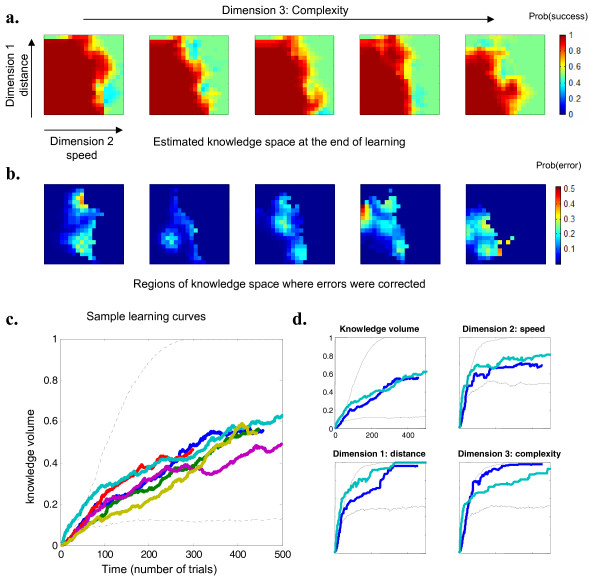
**Performance of the adaptive algorithm in tracking the knowledge of actual children**. a. Estimated knowledge space at the end of training for one subject (same format as figure 2a). This subject attained a high level of achievement in distance and complexity dimensions, but remained limited in the speed dimension. b. Regions of knowledge space where errors were corrected in the course of training. The graph shows the probability density of errors observed throughout the training period which were later corrected (i.e. at the end of training the corresponding region had an estimated probability of success > 0.95). c. Evolution of the knowledge volume for six representative children. All showed evidence of learning (compare figure 2b). d. Here we compare the evolution of knowledge for two children; measured in a narrow rectangular cube along each dimension, which allows a relatively bias free measurement of progress for that dimension in particular. Both children quickly hit an asymptote on the speed dimension, but their performance showed a double dissociation along the distance and complexity dimension. Note: The dotted curves in figures c and d are included for comparison and represent knowledge volume change over time in simulations with a fixed knowledge of 0.4 and 1 (as in figure 2b).

Secondly we examined the software data for evidence of progress in performance. The same Matlab programs that were used to analyze the simulation data were used to construct models of how the children's estimated knowledge space changed over time with utilization of the software (e.g. Figure 4a). All children showed evidence of progress using the software, as measured by an increase in knowledge volume (Figure 4c). Children's curves on this measure were similar to those from simulations with a moderate learning rate, and were considerably lower than simulations of children with a perfect fixed knowledge. This confirms empirically that the software challenged children. Furthermore, empirical evidence of learning could be seen, inasmuch as children showed progress in areas where they initially failed. For instance in Figure 3b we can see several locations within the learning space where a child initially made errors, but where performance was later almost perfect. Similar evidence can also be seen in a movie which shows the change in knowledge space over time for one particular child (additional file 1).

Finally, different children showed different profiles of performance and of knowledge acquisition, as evidenced by the progression at different rates on different difficulty dimensions. For example, in Figure 4d we can see a comparison between two children's performance on the distance and complexity dimensions, suggesting a double dissociation: One child progresses quickly on the distance dimension, but slowly on the complexity dimension, suggesting a need to increase understanding of symbolic numbers and/or fluency of elementary arithmetic, while the other child shows the converse pattern, suggesting a greater need to increase precision in the representation of quantities.

#### Observations

Our informal observations from the remediation sessions confirmed that children enjoyed using the software. Feedback from parents and teachers was also positive. Teachers reported that children's confidence in their mathematical ability improved, possibly a result of a new positive association with math created by the rewarding game. Aspects of the game which children found particularly rewarding and entertaining were the speed deadlines, the character animations, the sound feedback, and winning rewards and new characters.

Some problems in the software design were identified. The principal difficulty observed was that after about 10 hours of use (an average of 420 trials), children tended to become bored with the software. In the following section, we discuss why this might be the case, and possible methods to combat this effect.

## Discussion

Simulations showed that the algorithm correctly adapted to both simulated children's knowledge and learning rates, even when these were different across different dimensions. Results from real children were similar to simulation results, showing that children did learn in the process of using the software. Of course, in order to rigorously test the effectiveness of the software in remediation of dyscalculia, children's performance needs to be tested using independent pre and post tests. The accompanying paper [1] presents this methodology and analysis for the same group of children; thus we defer such discussion to it, and focus here on details relevant to the software design and future uses.

The potential for future uses and adaptations of the software is promising. Children who used the software showed different profiles of performance on different dimensions, suggesting that response to the intervention could be an interesting variable to investigate in the future with a larger sample. For instance, perhaps children with different subtypes of dyscalculia might perform better or worse on particular difficulty dimensions of the software. Children's response to the intervention could also be used in the future to investigate different theories of dyscalculia. As discussed earlier, our software design takes into account two possibilities as to the "core deficit" underlying dyscalculia; a) an impairment in number sense itself, and b) a impairment in the links between the quantity representation and the symbolic representations of number, without a direct deficit of number sense per se. Children in whom the first cause is dominant should benefit primarily from training on the distance dimension, while children with impaired symbol-quantity links should benefit primarily from training on the complexity dimension. Furthermore, with minimum changes to the complexity dimension, a purely non symbolic version of the software, training children solely to compare the numerosity of sets of dots, could be made. If children still showed a response to this version (using pre and post tests), this would support an impairment in non-symbolic number sense as the cause of a "core deficit".

Two software design problems were identified. First, for the age range tested (7–9), the initial levels of the software were too easy for most children, and the software took too long to adapt to their initial ability. An easy way to solve this problem in the future is to start the algorithm assuming a certain degree of prior knowledge for each child, so that it is able to progress to more difficult problems more quickly.

The second design problem was that some children became bored with the software after about 10 hours of use. This was not because they were performing at ceiling; on the contrary, not one child had reached ceiling performance on both the complexity and distance dimensions, and in fact the "speed" dimension of the software was programmed so that even adults would have difficulty in reaching ceiling performance. Instead, we believe several other factors may have contributed to this effect: the aforementioned initial slowness of the game to reach children's zone of proximal development (because it started at a very easy level), insufficient variation in the game, slowness of game play (especially on the "board screen"), and finally the fact that after about 10 hours of play, children had won all of the rewards and characters available, considerably reducing their motivation to continue using the software.

We have therefore developed a new version of the software, in which we have addressed all of these issues. We adjusted the algorithm so that it does not necessarily start at the easiest level, but instead can be preprogrammed to assume a moderate amount of prior knowledge (e.g. a cube of high probability of success ranging from 0 to 0.4 on all dimensions). Secondly, we included a second "graphical shell" or "game world", which uses the same underlying game logic with different settings, images and characters. This provides more variety and entertainment for children, and has the added bonus of doubling the number of rewards and characters that children can win. We also sped up automatic movement on the "board screen" making game play faster. Finally, in our current work, we now restrict the maximum age of children using the software to 5–8 years, as children of this age are more engaged by the software and found it more challenging.

The software still has limitations, and although it appears to produce improvement on some tasks (see companion paper [1]), it is unlikely to provide a simple "cure" for dyscalculia. The software currently focuses solely on a small range of numerical magnitudes (1–9). It is not clear whether training on single-digit numbers will transfer to larger numbers and to the concept of place value, which pose frequent difficulties to children with learning disabilities. In addition, there are many mathematical concepts (e.g. multiplication, division, fractions), which are not included in the software. However, there is of course the possibility of using the same software algorithm and framework for expansion into these higher arithmetic domains. In the future, new versions of the game could be made which might train multi-digit number sense, fractions, or multiplication facts.

## Conclusion

We have described in this paper the conception and development of adaptive software for the remediation of dyscalculia, including full specifications of the learning algorithm used, and testing of the software; both using mathematical simulation and with a group of children with mathematical learning difficulties. The design of the software incorporated four major principles: Enhancing number sense, cementing links between symbolic and non-symbolic representations of number, conceptualizing and automatizing arithmetic, and maximizing motivation. These principles were implemented in the software by the use of three adaptive dimensions (distance, speed and conceptual complexity), which together form a multidimensional learning space.

A multidimensional learning algorithm was used to model and adapt to children's performance in this learning space. Simulations of the algorithm's performance showed that it was able to accurately model children's knowledge, and to respond differently to different initial knowledge levels and learning rates. The algorithm was validated in a small sample of 7–9 year old children who had mathematical learning difficulties. It successfully adapted to children's performance (including to their individual difficulties) and kept children's mean success at close to the desired rate. Children's learning could be seen as an increase in knowledge volume, and as eventual success in areas of initial failure.

These results, along with the results from pre and post testing (see accompanying paper [1]), suggest that the software may be useful for remediation of dyscalculia, at least for children aged 7–8 and under. In addition, there is potential to use the software to investigate different causes and subtypes of dyscalculia. Although the current paper focuses on dyscalculia, we emphasize that the learning algorithm developed is a general one, which could be used in any domain. Additionally, the software may have applications to the general instruction of number sense for normal children at a younger age (e.g. 3–6 yrs). Finally, the software described is open source and available online for free download, thus allowing other research groups as well as the general public to use and test its ability to help children at risk for mathematical learning disabilities.

## Competing interests

The author(s) declare that they have no competing interests.

## Authors' contributions

The adaptive software was designed by AJW and SD (with intellectual input from LC and DC), and programmed by AJW. Most game graphics were designed by PP. Simulations of the software were carried out by SD and AJW. Collection and analysis of data from children was carried out by AJW and SKR, with assistance from DC, SD and LC. All authors read and approved the final manuscript, which was prepared by AJW and SD.

## Supplementary Material

Additional File 1 Example knowledge space movie. This movie file, output from our Matlab analysis program, shows the progress in knowledge space over time of one child using the software, shown as ten "slices" through the three-dimensional knowledge space cube. The x axis is speed, the y axis distance, and the z axis complexity. Red represents high probability of success, blue high probability of failure, green background = chance level (50%). As the child makes errors, patches of blue appear. We can see particular areas of difficulty which are consistently blue (e.g. for this child the speed dimension and higher levels of the complexity dimension), however we see that over time many of these become red as the child makes progress. Click here for file
